# INTRAGASTRIC BALLOON AND IMPACT ON WEIGHT LOSS: EXPERIENCE IN QUITO, EQUADOR

**DOI:** 10.1590/0102-672020230002e1731

**Published:** 2023-05-26

**Authors:** Jonathan Jerez, David Cabrera, Carlos Cisneros, Monica Moreno, Daniela Guaitara, Chiristian Benavides, Martha Fors, Kirsten Falcon

**Affiliations:** 1 SOM Clinic, Gastroenterology – Quito, Equador; 2 Universidad de las Américas, Faculty of Health Sciences – Quito, Equador;; 3 Universidade de São Paulo – São Paulo (SP), Brazil.

**Keywords:** Endoscopy, Body mass index, Digestive system, Body weight, Obesity, Endoscopia, Índice de Massa Corporal, Sistema Digestório, Peso Corporal, Obesidade

## Abstract

**BACKGROUND:**

Obesity is associated with different medical conditions, such as cardiologic, respiratory, gastrointestinal, and genitourinary, and constitutes a severe health problem.

**AIMS:**

This study aimed to evaluate the use of intragastric fluid-filled balloon in the reduction of weight and other measurements related to body composition.

**METHODS:**

This is a retrospective, monocentric study involving all patients who opted for the intragastric balloon Spatz^®^ placement from January 2018 to July 2019, with fulfillment of inclusion and exclusion criteria. The patients were analyzed after 6 and 12 months after the intragastric fluid-filled balloon placed.

**RESULTS:**

A total of 121 subjects were included in this study, with 83 (68.6%) females and 38 (31.4%) males. The mean age was 36 years and height was 1.64±0.09. Weight mean and standard deviation was 89.85±14.65 kg, and body mass index was 33.05±4.03; body mass index decreased to 29.4 kg/m^2^ with a mean weight of 79.83 kg, after 12 months of follow-up. There were statistical differences between body mass index and the 12 months in fat percentage, fat-free mass (kg), visceral fat area, and basal metabolic rate. There was a significant variation according to gender, with males having highest reduction. The percentage of excess weight loss was 46.19, and the total weight loss was 9.24 at the end of the study.

**CONCLUSIONS:**

The study demonstrated a benefit of intragastric fluid-filled balloon on weight loss after 12 months. At the end of treatment, body mass index and the measurements of body composition were significantly lower. Men benefited more than women from the treatment.

## RESUMO

**RACIONAL:**

A obesidade está associada a diferentes condições médicas, tais como cardiológicas, respiratórias, gastrointestinais, geniturinárias entre outras e constituem um grave problema de saúde.

**OBJETIVOS:**

Avaliar o emprêgo do balão intragástrico na redução de peso e em outras medidas relacionadas à composição corporal.

**MÉTODOS:**

Estudo retrospectivo, monocêntrico, incluindo todos os pacientes que optaram pela colocação de balão intragástrico Spatz^®^ entre janeiro de 2018 e julho de 2019, com cumprimento dos critérios de inclusão e exclusão.

**RESULTADOS:**

Cento e vinte e um indivíduos foram recrutados neste estudo. A média de idade foi de 36 anos e estatura de 1,64±0,09, sendo 83 (68,6%) do sexo feminino e 38 (31,4%) do sexo masculino. A média do peso e o desvio padrão foram de 89,85±14,65 kg e o índice de massa corporal foi de 33,05±4,03. Após 12 meses, o índice de massa corporal diminuiu para 29,4 kg/m^2^ com um peso médio de 79,83 kg. Foram registradas diferenças estatísticas no índice de massa corporal, no percentual de gordura, massa livre de gordura (kg), área de gordura visceral e taxa metabólica basal. Houve variação significativa de acordo com o sexo, sendo o masculino com maior redução. O porcentual de perda de excesso de peso foi de 46,19% e de perda de peso total de 9,24 %ao final do estudo.

**CONCLUSÕES:**

O estudo demonstrou benefícios do balão intragástrico na perda de peso após 12 meses de colocação do balão. Ao final do tratamento, o índice de massa corporal e as medidas de composição corporal foram significativamente menores. Os homens se beneficiaram mais do que as mulheres do tratamento.

## INTRODUCTION

Overweight and obesity remain a big public health issue, affecting over one-third of the world's population today^25^. According to the World Health Organization (WHO), obesity is “a condition in which percentage body fat (PBF) is increased to an extent in which health and well-being are impaired”, and, due to the alarming prevalence increase, it was declared as a “global epidemic”^27^.

In 2016, 1.3 billion adults were overweight worldwide and the number of adults with obesity increased by sixfold from 100 to 671 million (69–390 million women and 31–281 million men) between 1975 and 2016^24^. One of the main challenges in addressing overweight and obesity lies in adopting a common public health measure of these conditions^28^. As a result, body mass index (BMI) is adopted as an indicator for defining overweight and obesity^23,35^.

In Ecuador, according to data published in the National Health and Nutrition Survey Ecuador (ENSANUT-ECU 2011–2013)^22^, the prevalence of overweight and obesity at a national level in people older than 19 years is approximately 62.8%, with rate being greater in women (65.5%) than in men (60%).

Currently, there are many treatments available for adults with overweight and obesity, including reduced calorie diet, exercises, behavior modification, and use of specific treatments; however, some of these approaches do not achieve very good results. The intragastric balloon (IGB) is a reversible, endoscopically placed device approved for limited-term use in overweight and obese patients. Since bariatric surgery is of high cost, with complication risks and invasiveness, the use of IGB treatment is a good alternative as it is safer and of lower costs. Some studies have shown moderate weight loss of 15 kg or more with the use of the IGB^21,30,36^.

This technique has been the most frequently used in practice and the most studied for this medical condition and may be performed in patients with mild obesity (BMI=30 kg/m^2^). Body weight loss achieved with intragastric balloon placement is associated with improvements in obesity-related metabolic illness^18^. Its placement also affects hunger control and gastric emptying through alterations in gut hormones and peptides^7^.

A meta-analysis showed that endoscopic obesity treatment could be effective and of substantial value if combined with a multidisciplinary and comprehensive treatment plan^5^.

The objective of this study was to contribute to the experience in the country in the evaluation of the use of IGB for achieving weight loss and its impact on body composition measurements.

## METHODS

This is a retrospective, monocentric study, which included all patients who opted for the intragastric balloon Spatz® placement from January 2018 to July 2019, with fulfillment of inclusion and exclusion criteria.

The inclusion criteria for this study were as follows: patients who opted for the Spatz® intragastric balloon whose clinical history contained complete data for 12 months in the period between January 2018 and July 2019. A total of 121 patients were selected. Patients who do not complete 12 months of treatments were excluded.

The patients were analyzed after 6 and 12 months of the IGB placement, based on the parameters such as body weight (kg), BMI (kg/m^2^), musculoskeletal mass, percentage fat mass (%), fat-free mass (kg), basal metabolic rate (kcal), visceral fat area (cm^2^). and phase angle (°).

### Statistical analysis

Qualitative variables are presented as proportions and percentages and continuous variables as mean and standard deviation. The normal distribution of continuous variables was explored using the Shapiro-Wilk test and found that most of them were non-normally distributed.

For body composition variables, statistical differences between gender were calculated with Mann-Whitney U test. Changes of body composition variables were calculated by each variable at last visit (12 months) minus variables at baseline. Comparisons among means of continuous variables were made using Friedman test and non-parametric alternative to the one-way ANOVA with repeated measures when data are non-normal. Post-hoc Dunn's test was performed. The significance was set at 0.05 (p<0.05). Mann-Whitney U test was used to compare the means of 2 independent samples, and the Wilcoxon test to compare the means of paired samples. Paired t-tests were used to compare mean of angle phase at 6 and 12 months. The relationship between body composition indexes and gender was analyzed using Pearson's chi-square test. Spearman's correlation coefficient was used to determine the correlations between variations in weight and body composition parameters. The data were analyzed by SPSS (version 24.0; SPSS Inc., Chicago, Illinois, USA).

### Ethical aspects

The participants were informed about the purpose of the procedure. They first read over and sign a consent form informing them of their rights and the benefits and risks associated with the placement of intragastric balloon. All procedures performed were in accordance with the ethical standards of the institutional and with Helsinki Declaration. This report followed STROBE guidelines for observational studies. The study was approved by Ethics Committee SOM-2017-003.

## RESULTS

A total number of 121 patients were selected for analysis, with 83 (68.6%) females and 38 (31.4%) males. Patients had a mean age of 36 years and height of 1.6 m±1 cm. Weight mean and standard deviation was 89.8±14.6 kg, and the BMI was 33.0±4.0 at pretreatment. At the end of the treatment, the BMI decreased to 29.4 kg/m^2^ with a mean weight of 79.8 kg. Compared to baseline values, the patients experienced significant reductions in weight, BMI, fat-free mass (FFM) (kg), basal metabolic rate (BMR) (kcal), visceral fat area (VFA), and phase angle (p<0.00) (Table 1).

**Table 1:** Results of 12 months of intragastric fluid-filled balloon treatment (n=121)

Characteristics	Average (mean±SD)	Average (mean±SD)	Average (mean±SD)	p-value*
	pretreatment	6 months	12 months	
Body weight (kg)	89.8±14.6	81.4 ±13.4	79.8±16.0	<0.00
BMI (kg/m^2^)	33.0±4.0	29.8±3.3	29.8±3.5	<0.00
Musculoskeletal mass	28.9±7.0	27.4±6.7	27.4 ±6.7	0.22
Percentage fat mass (%)	42.3±6.7	39.2±7.5	39.0±7.7	<0.00
Fat-free mass (kg)	37.8±7.9	31.9±7.9	31.9±7.9	<0.00
Basal metabolic rate (kcal)	1491.1±249.7	1447.8±241.4	1441.6±239.0	<0.00
Visceral fat area (cm^2^)	181.3±205.2	180.7±38.9	154.6±43.9	0.02
Phase angle (°)	5.5±0.8	–	5.6±0.7	<0.00**

* Friedman test

** Student's t-test (normally distributed variable). BMI: body mass index; SD: standard deviation; IGB: intragastric fluid-filled balloon

The differences between pretreatment (baseline) values and after the periods of follow-up (final) are displayed in (Figure 1 A-1F).

**Figure 1A:**
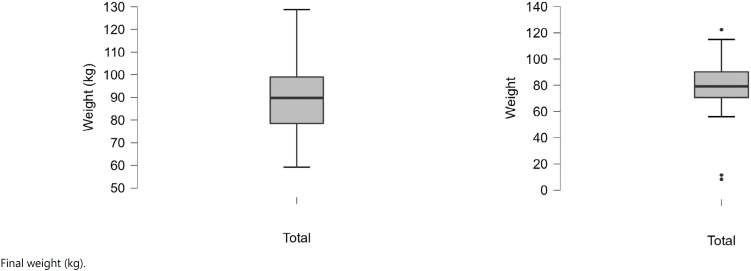
Baseline and final weight (kg).

**Figure 1B:**
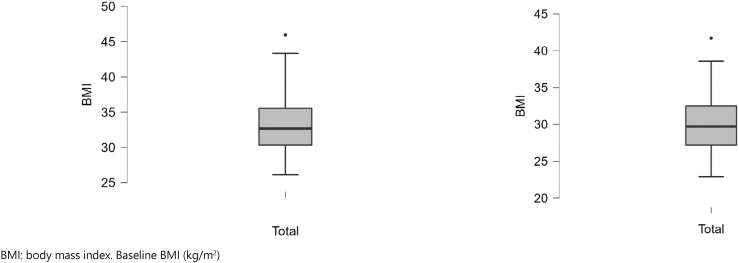
Baseline and final BMI (kg/m^2^).

**Figure 1C:**
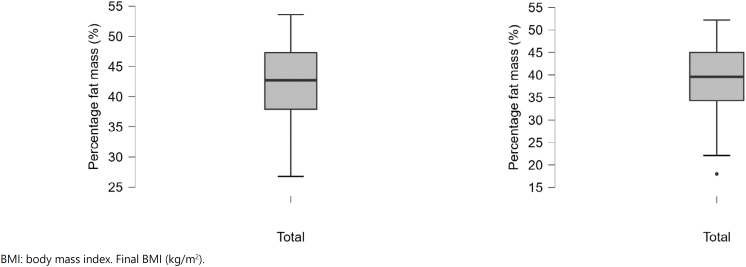
Baseline and final percentage fat mass (%).

**Figure 1D:**
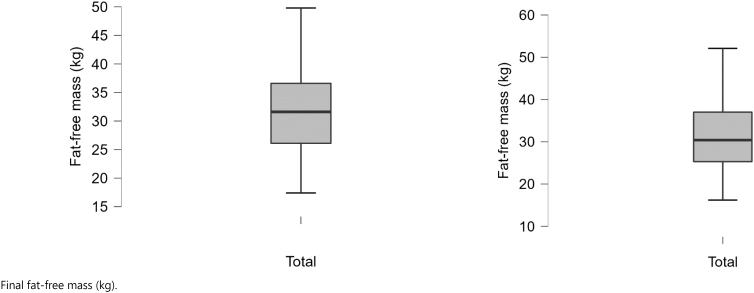
Baseline and final fat-free mass (kg).

**Figure 1E:**
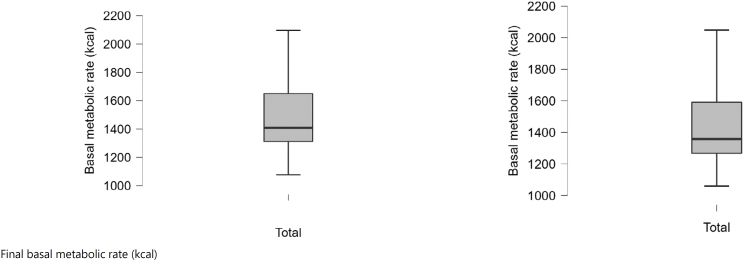
Baseline and final basal metabolic rate (kcal).

**Figure 1F:**
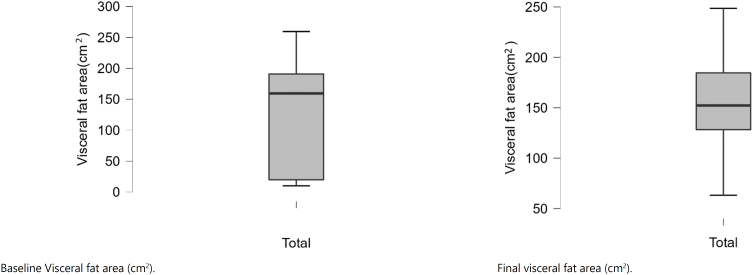
Baseline and final visceral fat area (cm^2^).

The post-hoc comparison with Dunn's test showed a significant mean difference in indicators, before the IGB placement and after 6 and 12 months. In addition, a non-significant statistical difference is found between 6 and 12 months (Table 2).

**Table 2:** Post-hoc analysis of mean differences.

		Time points	Mean differences	p-value*	95% Confidence interval	
					Lower bound	Upper bound
Weight						
	Pretreatment	6 months	8.4	0.00	3.8	12.9
		12 months	10.0	0.00	5.4	14.5
	6 months	12 months	1.5	1.00	-2.9	6.1
BMI						
	Pretreatment	6 months	3.46	0.00	2.25	4.67
		12 months	3.20	0.00	1.99	4.41
	6 months	12 months	0.25	1.00	-0.95	1.47
Percentage fat mass (%)						
	Pretreatment	6 months	3.16	0.00	0.88	5.45
		12 months	3.32	0.00	1.03	5.60
	6 months	12 months	0.15	1.00	-2.12	2.44
Fat-free mass (kg)						
	Pretreatment	6 months	5.93	0.00	3.47	8.39
		12 months	6.23	0.00	3.77	8.69
	6 months	12 months	0.30	1.00	-2.15	2.76
Visceral fat area (cm^2^)						
	Pretreatment	6 months	-45.38	0.02	-85.77	-5.00
		12 months	-23.12	0.50	-63.50	17.26
	6 months	12 months	22.26	0.55	-18.12	62.65

* Dunn's post-hoc analysis. BMI: body mass index.

Average weight loss in the male group was 14.7±20.7 kg, which was higher than that in the female group. After 6–12 months of follow-up, our patients showed a mean BMI of 3.4 kg in men and 3.1 kg in women, but we could not demonstrate a significant difference between gender. A statistically significant difference was observed only in BW, MME, FFM, and phase angle. Values in men were higher (Table 3).

**Table 3:** Mean differences according to sex of patients at 12 months of balloon placement.

Mean differences	Male n=38 (mean ± SD)	Female n=83 (mean ± SD)	p-value*
Body weight (kg)	14.7±20.7	7.8±6.1	0.03
BMI (kg/m^2^)	3.4±2.1	3.1±2.4	0.39
Musculoskeletal mass	1.7±1.5	1.3±1.3	0.05
Percentage fat mass (%)	4.3±3.7	2.8±2.9	0.05
Fat-free mass (kg)	7.3±5.3	5.7± 4.5	0.15
Visceral fat area (cm^2^)	10.8±70.2	28.7±84.0	0.17
Phase angle (°)	6.3±0.8	5.3±0.4	0.00

* Mann-Whitney U test. BMI: body mass index; SD: standard deviation.

The mean percentage of total weight loss (TWL) achieved was 9.24±5.71 at 6 months and 9.37±6.51 at 12 months (non-significant difference: p=0.73, p>0.05). The mean percentage of excess weight loss (EWL) was 46.19±38.51 at 6 months and 45.28±38.43 at 12 months (p=0.72, p>0.05). We did not find any statistical differences between gender in these indicators in any of the two times (Table 4).

**Table 4:** Means of percentage of total weight loss and percentage of excess weight loss according to gender, at 6 and 12 months follow-up.

Mean (%)	6 months		p-value	12 months		p-value*
	Male	Female		Male	Female	
TWL	10.54±6.385	8.64±5.31	0.09	10.41±6.41	8.89±6.54	0.23
EWL	54.25±40.34	42.0±0.37	0.12	43.71±39.64	43.78±39.64	0.50

* Mann-Whitney U test.

Regarding weight loss results, a generally accepted criterion to know if the method is successful is if percentage of TWL is >7% and percentage of EWL is >30%. According to this criterion, we compared percentage of EWL at two times. The proportions of patients having successful weight loss (TWL>7%) were 64.0% (n=78) at 6 months and 62.0% (75) at 12 months. The proportions of patients having successful weight loss (EWL>30%) were 62 and 62.8% at 6 and 12 months, respectively. There was a statistically significant difference in both cases. A statistically meaningful linear correlation between a 6-month EWL and TWL and a 12-month EWL or TWL was found (Table 5).

**Table 5:** Differences between 6 months and final measurements of percentage of total weight loss (%) and excess weight loss (%).

		6 months		12 months		p-value*
		No.	%	No.	%	
TWL (%)						
	TWL>7%	78	64.5	75	62.0	0.00
	TWL<7%	43	35.5	46	38.0	
EWL (%)						
	EWL>30%	75	62.0	76	62.8	0.00
	EWL<30%	46	38.0	45	37.2	

* χ^2^

** Pearson's correlation coefficient.

## DISCUSSION

This is the first study to document outcomes with the use of IGB therapy in Ecuador. In the current study, 6 months of treatment with IGB was associated with improvements in the indicators measured. At the end of 12 months term, significant reductions were seen in both weight and BMI for most of the cohort. Some studies have reported on the efficacy of IGB in inducing significant weight loss over the short to medium period^16,26,29^.

In our study, mean weight loss 12 months after balloon placement was 14.71±20.71 in men and 7.87±6.19 in women. At the end of the IGB treatment period, it showed significant weight loss. Many authors have reported figures ranging from 9.5 to 20.1 kg^13,14,20,31,32^.

After 6–12 months of follow-up, our patients showed a mean weight loss of 8.25 kg, similar to other studies evaluating IGB^8–10^. In the current study, statistically significant differences were observed between gender. Al-Sabah et al., for example, did not report significant variation in the weight loss according to this variable^3^.

Some studies reported that there was a significant reduction of visceral fat area^6,34^, while other authors reported that the visceral fat area showed no significant decrease^33^.

Sekino et al.^33^ also reported that preoperative intragastric balloon therapy may produce a favorable reduction of the visceral fat area and that the use of IGB for a few weeks may serve as a useful preparation procedure prior to laparoscopic bariatric surgery. We found that initial value of VFA decreased along the period of study.

Our results show a significant decrease in percentage of fat mass and free fat mass, while Donadio et al.^11^ found a reduction in percentage of fat mass (-19.5%), but not in fat-free mass.

Regarding FFM, our study demonstrated that men had a reduction of 7% and women 5%, without statistical differences between them. In a study performed in Poland, there was a 5.4% reduction in FFM^15^. Folini et al.^12^ also reported a decrease in FFM and percentage of fat mass. Another study reported a decrease in FFM at 6–12 months^31^.

These results were better observed in our patients between pretreatment and 6 months, where the reduction of these indicators was higher. Their means were not different between 6 and 12 months.

The overall TWL and EWL were 9.37±6.51% and 45.28±38.43% at 12 months, respectively. In the current study, approximately 60% of the individuals had very good results at 6 and at 12 months in both indicators, taking into account the criteria of Abu et al.^1^.

Agnihotri et al.^2^ found a higher percentage of TWL at 12 months (14.7±11.8%) and reported that 60.4% of patients achieved more than 10% of TBWL and 55.4% had more than 25% of EWL. Even in a different cutoff point, our results are found very similar.

Al-Subaie et al.^4^ reported %TBWL of 10.44% and EWL (%) 40.84%, which are very similar to our results. According to Lewis et al.^19^, a 10% loss in body weight (10%TWL) will translate into a reduction of visceral, central, and abdominal fat, as well as the size of the liver.

Excess weight loss of 38.5% was the results for the study by Al-Sabah et al.^3^, which is lower than the values in the current study, while Guedes et al.^17^ reported percentage of EWL of 56.04±4.90, which is higher than ours. Al-Sabah et al.^3^ also found statistically significant differences between gender regarding percentage of EWL, while we did not^35^.

## CONCLUSION

The study demonstrated a benefit of intragastric fluid-filled balloon on weight loss after 12 months. At the end of treatment, BMI and the measurements of body composition were significantly lower. Men benefited more than women from the treatment.
